# Coronary heart disease is associated with nonalcoholic fatty liver disease in patients without hypertension and diabetes

**DOI:** 10.1097/MD.0000000000020898

**Published:** 2020-06-26

**Authors:** Zipeng Liu, Rufeng Wei, Yan Li

**Affiliations:** Department of Ultrasound Diagnosis, Hanzhong Central Hospital, Shaanxi, China.

**Keywords:** coronary heart disease, Gensini score, nonalcoholic fatty liver disease, ultrasound

## Abstract

This study was performed to explore the relationship between coronary heart disease (CHD) and nonalcoholic fatty liver disease (NAFLD) in patients without hypertension and diabetes with a focus on predicting CHD.

In total, 78 consecutive patients without hypertension and diabetes who were suspected of CHD underwent coronary angiography (CAG) or computed tomography CAG. They were segregated into the CHD and non-CHD group according to the CAG or computed tomography angiography results. The Gensini score was calculated based on CAG results in the CHD group. All patients underwent ultrasonographic measurement of the liver, subcutaneous fat, and visceral fat thickness.

The CHD and the Gensini score were significantly correlated with V1, V2, and NAFLD. As the grade of NAFLD increases, the Gensini score was increased. After correcting for confounding factors, NAFLD (*B* = 2.474, *P* < .001, 95% confidence interval: 3.32–42.406) and cholesterol (*B* = 1.176, *P* = 0.025, 95% confidence interval: 1.155–9.101) were predictor for CHD.

The CHD is associated with NAFLD in the patients without hypertension and diabetes. The high-grade NAFLD may be predicted the risk of CHD in patients without hypertension and diabetes.

## 1 Introduction

Studies have shown that obesity is an independent risk factor for coronary heart disease (CHD), especially the visceral adipose tissue (VAT).^[1]^The excess of fat deposition in VAT has been associated with increased mortality and morbidity risks.^[2]^ VAT has a proinflammatory profile, favoring the development of CHD and metabolic diseases.^[3]^ As a consequence, patients’ liver is exposed to a high concentration of free fatty acids throughout the portal vein circulation, thus causing an alteration of its metabolism which resulting the nonalcoholic fatty liver disease (NAFLD).^[4]^

The NAFLD is a clinical pathologic syndrome that progresses from simple steatosis to cirrhosis. NAFLD has gradually become the most common cause of chronic liver disease worldwide.^[5–7]^ Previous studies have indicated that NAFLD is associated with subclinical atherosclerosis as indicated by an increased carotid intima-media thickness and greater arterial stiffness.^[8]^ Cross-sectional studies have indicated that NAFLD is associated with an increased prevalence of cardiovascular disease.^[9,10]^ However, whether the presence of NAFLD predicts the likelihood of a CHD event or whether patients with severe NAFLD have a greater likelihood of cardiovascular disease events remains uncertain. Therefore, we explored the relationship between NAFLD and CHD in patients without hypertension and diabetes.

## 2 Materials and methods

### 2.1 Patients

This study included 78 patients (50 men, 28 women; age, 25–68 years) who presented to the cardiovascular department of our hospital from June to December 2016. Patients were divided into CHD group and non-CHD group according to the results of coronary angiography.^[11]^ The inclusion criterion was first and initial suspected CHD. The exclusion criteria were a serum creatinine concentration of >17.68 μmol/L, class III or IV heart failure, hypertension, diabetes, chronic inflammatory disease, stroke, myocardial infarction, congenital heart disease, known hypersensitivity to iodine-based contrast agents, chronic liver disease, and hypothyroidism.

Dyslipidemia was defined as a total serum cholesterol concentration of ≥12.22 mmol/L, low-density lipoprotein cholesterol (LDL-C) concentration of ≥7.9 mmol/L, serum triglyceride concentration of ≥8.3 mmol/L, and serum high-density lipoprotein cholesterol (HDL-C) concentration of <2.2 mmol/L and/or current use of antihyperlipidemic medications. Smoking was defined as past or current smoking; nonsmoking was defined as never having smoked.

All patients provided written informed consent after they received advice regarding the potential risks of radiation exposure, the administration of iodine-containing contrast agents, and coronary angiography. This study was approved by the Hospital Ethics Committee.

### 2.2 Anthropometric measurements

The body mass index was calculated as weight (kg) divided by height squared (m^2^). Waist circumference was measured at the anatomic waistline, which is located at the natural indentation between the iliac crest and the 10th rib.

### 2.3 Nonalcoholic fatty liver disease

We used ultrasound to evaluate NAFLD. Ultrasonographic evaluations were performed by 2 physicians with more than 5 years of experience each, and both were unaware of the aims of the study. We used a Doppler ultrasound system (EPIQ 5; Philips, Bothell, WA) with a 3.5-MHz probe. We obtained a sagittal view of the right lobe of the liver and right kidney, a transverse view of the left lateral segment of the liver and spleen, a transverse view of the liver and pancreas, and any focal areas of altered echotexture. The severity of echogenicity was graded as follows: grade 0, normal echogenicity; grade 1, slight diffuse increase in fine echoes in liver parenchyma with normal visualization of diaphragm and intrahepatic vessel borders; grade 2, moderate diffuse increase in fine echoes with slightly impaired visualization of intrahepatic vessels and diaphragm; and grade 3, marked increase in fine echoes with poor or absent visualization of the intrahepatic vessel borders, diaphragm, and posterior right lobe of the liver.^[12]^

### 2.4 Subcutaneous fat and visceral fat thickness measurements

We used the same Doppler ultrasound system with a 3.5-MHz probe to measure the subcutaneous fat.^[13]^ The patients underwent ultrasonographic fat measurements the morning after an overnight fast. The probe was used to perform a cross-sectional scan and placed at the midpoint between the xiphoid process and the umbilicus. The distance between the skin and subcutaneous fat interface to the linea alba was designated S1. The probe was moved to the right edge of the rectus abdominis. The distance between the skin and subcutaneous fat interface to the leading edge of the external oblique muscle was designated S2. A 7.4-MHz probe was used to measure the visceral fat. The distance from the trailing edge of the linea alba to the anterior wall of the abdominal aorta was designated V1. The distance from the posterior edge of the external oblique muscle to the right edge of the spine was designated V2.

Ultrasonographic measurements were performed by 2 physicians with more than 5 years of experience each, and both were unaware of the aims of the study.

### 2.5 Blood parameter measurements

Venous blood was collected from all patients on the morning of the 2nd day, after fasting for 8 hours. The blood parameters measured were the cholesterol, HDL-C, LDL-C, and myocardial enzyme concentrations as well as liver function parameters.

### 2.6 Statistical analysis

Data analyses were performed using IBM SPSS Statistics for Windows, Version 20.0 (IBM Corp, Armonk, NY). All descriptive data are expressed as mean ± standard deviation, median and interquartile range (25th–75th percentiles), or percent. The parameters were compared between the 2 groups using Student unpaired *t* test, Mann–Whitney *U* test, or the Chi-squared test where appropriate. Correlations between 2 continuous variables were examined using linear regression analysis. Correlations between 2 noncontinuous variables and non-normally distributed data were examined using Spearman regression analysis. Binary regression analysis was used to determine the predictors of CHD, adjusting for clinical risk factors including age, male sex, smoking habits, cholesterol, fat thickness, and fatty liver. A *P*-value of <.05 was considered significant.

## 3 Results

### 3.1 General characteristics

The patients’ general characteristics according to their CHD status are shown in Table 1. The mean age and number of male patients tended to be higher in the CHD than non-CHD group. The numbers of history of smoking were significantly different between the 2 groups. The cholesterol and LDL-C levels were higher in the CHD than non-CHD group. The visceral fat (V1, V2) and subcutaneous fat thickness (S1, S2) were significantly higher in the CHD than non-CHD group. Otherwise, the number of patients with NAFLD was higher in the CHD than non-CHD group, especially patients with moderate and severe grade NAFLD.

**Table 1:**
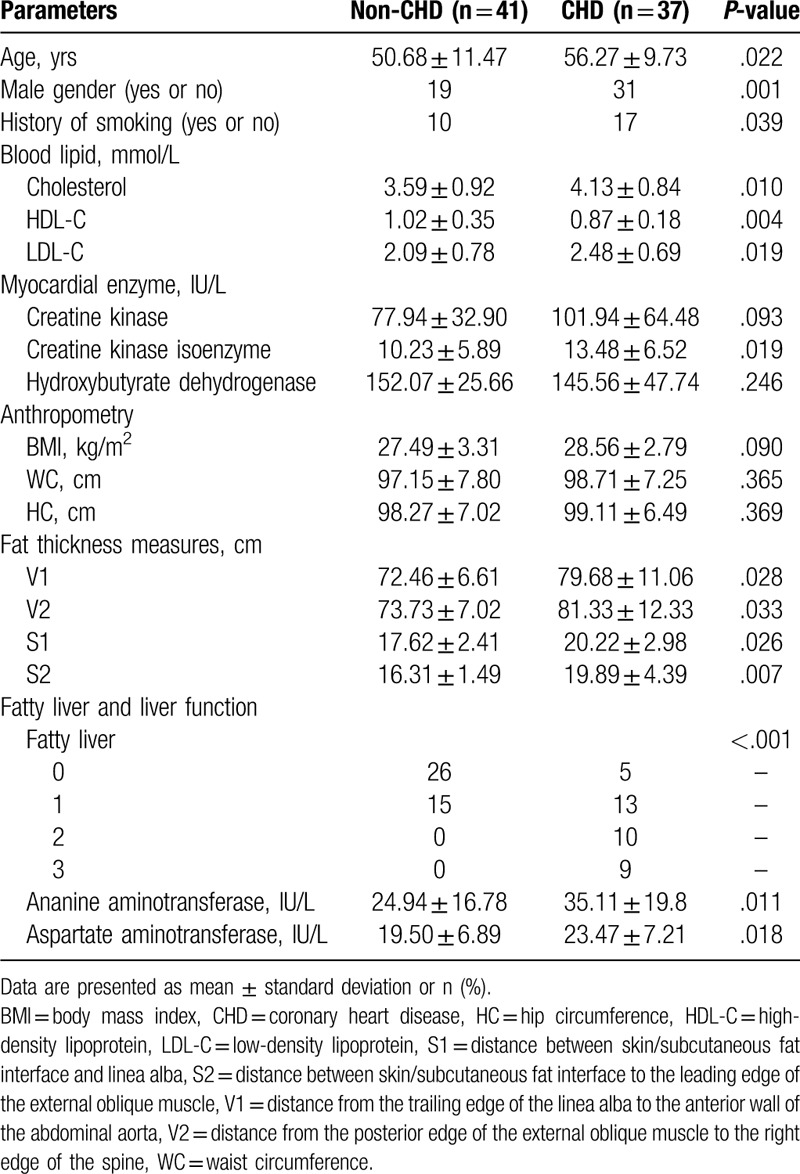
Patient characteristics vs CAD status.

### 3.2 Correlation analysis of CHD and Gensini score

Bivariate correlation analysis was used to estimate the relationship between a series of indicators and CHD/Gensini score (Table 2). CHD was significantly correlated with age, number of male patients, history of smoking, cholesterol, HDL-C, and LDL-C. Although whole-body parameters (body mass index) were not correlated with CHD, some fat thickness measures (V1, V2, S1, and S2) were correlated with CHD. NAFLD and liver function parameters (alanine aminotransferase and aspartate aminotransferase) were correlated with CHD. The Gensini score was significantly correlated with V1, V2, and NAFLD. As the grade of NAFLD increases, the Gensini score also increases (grade 0: 26.40 ± 17.47; grade 1: 29.54 ± 16.96; grade 2: 46.50 ± 23.27; grade 3: 101.11 ± 30.22).

**Table 2:**
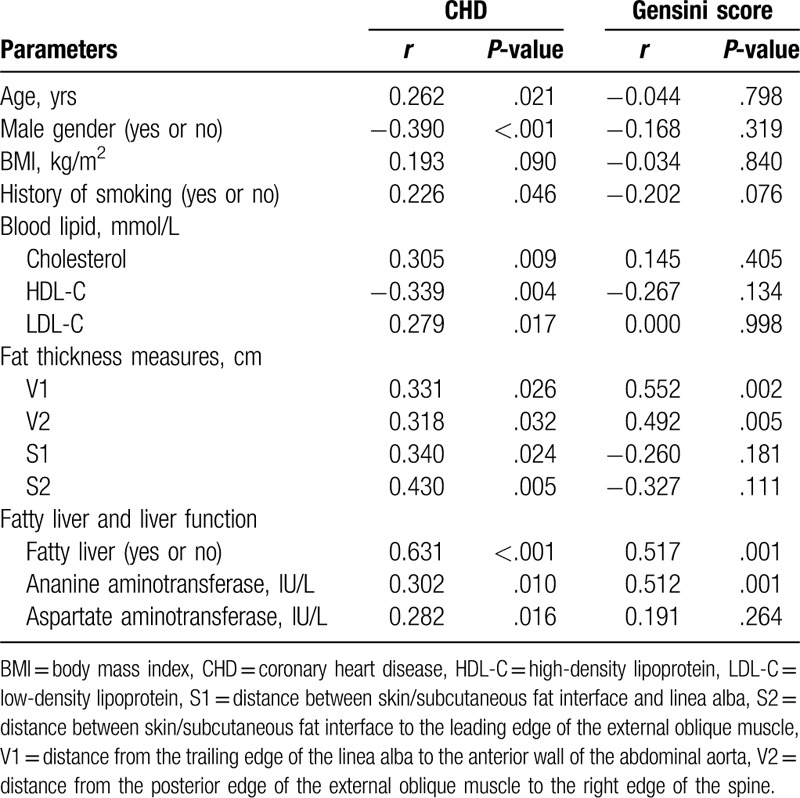
Correlation analysis of CHD and Gensini score.

### 3.3 Binary regression of CHD

We determined the impact of individual covariates on CHD using a binary regression model. After adjusting for age, number of male patients, history of smoking, cholesterol, HDL-C, LDL-C, and fat thickness, we found that cholesterol and NAFLD were risk factors for CHD (Table 3).

**Table 3:**
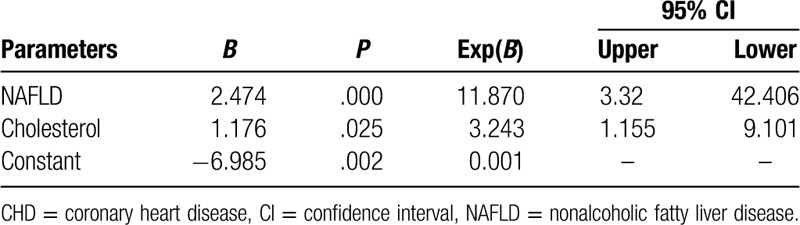
Binary regression of CHD.

## 4 Discussion

In this study, we explored the relationship between NAFLD and CHD in patients without hypertension and diabetes.

The study population was characterized by no history of hypertension and diabetes. We explored the relationship between NAFLD and CHD, based on the absence of hypertension and diabetes. Patients in the CHD group also had higher cholesterol and LDL-C levels. The visceral fat thickness and subcutaneous fat thickness in the CHD group was significantly thicker than that in the non-CHD group. CHD was significantly associated with NAFLD. Even after the logistic regression analysis that included the risk factors for CHD and the components of traditional CHD risk factors, the correlation between the existence of NAFLD and the determination of CHD remained significant. NAFLD might be a risk factor for the development of CHD. This is consistent with previous research results. Wong et al^[14]^ reported that CHD was found in 85% of patients with steatosis detected by ultrasound compared with 64% of patients without steatosis (*P* < .001). Liver steatosis remained an independent risk factor for CHD even after adjusting for demographic and metabolic parameters (odds ratio, 2.31).^[14]^

Our study also showed that the Gensini score was significantly associated with visceral fat thickness and NAFLD. As the grade of NAFLD increases and the thicker of the visceral fat, the Gensini score also increases. Several studies have shown an association of NAFLD with coronary artery wall calcification.^[15–17]^ In another study, the association of NAFLD and coronary artery wall calcification was independent of age, sex, and diabetes mellitus.^[8]^ Fat tissue is considered a metabolically active endocrine organ that produces proinflammatory cytokines, including interleukin,^[18]^ tumor necrosis factor α.^[19]^ Dysregulated secretion of proinflammatory and anti-inflammatory cytokines alongside increased oxidative stress, lipotoxicity of fatty acids, and systemic inflammation also plays a role in the development of liver steatosis.^[20–22]^ The metabolic abnormalities and associated changes in secretion of cytokines, increased oxidative stress, and systemic inflammation may provide a pathophysiologic link explaining the association of NAFLD with advanced coronary atherosclerosis.

After adjusting for age, number of male patients, history of smoking, HDL-C, LDL-C and fat thickness, we found that cholesterol and NAFLD were risk factors for CHD. Therefore, for patients without diabetes and hypertension, we could conclude that the higher grade of NAFLD, the higher cholesterol level and the more risk of CHD.

### 4.1 Limitations

This study has a few limitations. First, this clinical study was cross-sectional in design, and it was conducted at a single center with a relatively small number of patients. Second, the results need to be confirmed in a larger study. Finally, the gold standard technique for the diagnosis of NAFLD is biopsy. In our study, we used ultrasonography to diagnose NAFLD; therefore, our results need to be confirmed in future studies.

## 5 Conclusion

In the absence of hypertension and diabetes, NAFLD is associated with CHD. The high-grade NAFLD may be the predictor of CHD in patients without hypertension and diabetes.

## Acknowledgment

The authors thank Angela Morben, DVM, ELS, from Liwen Bianji, Edanz Editing China (www.liwenbianji.cn/ac), for editing the English text of a draft of this manuscript.

## Author contributions

Zipeng Liu and Yan Li wrote the main manuscript text. Rufeng Wei prepared Tables 1–3. All authors reviewed the manuscript.
